# The Impact of Medical Explainable Artificial Intelligence on Nurses' Innovation Behaviour: A Structural Equation Modelling Approach

**DOI:** 10.1155/2024/8885760

**Published:** 2024-09-26

**Authors:** Xianmiao Li, Qilin Zong, Mengting Cheng

**Affiliations:** ^1^ School of Economics and Management Anhui University of Science & Technology, Huainan, China; ^2^ School of Economics and Management Nanjing University of Aeronautics and Astronautics, Nanjing, China

**Keywords:** AI anxiety, AI self-efficacy, medical explainable artificial intelligence, nurses' innovation behaviour, organizational ethical climate

## Abstract

**Aim:** This study aims to investigate the influence of medical explainable artificial intelligence (XAI) on the innovation behaviour of nurses, as well as explore the dual-pathway mediating effect of AI self-efficacy and AI anxiety and organizational ethical climate as the moderating effect.

**Background:** To address the practical application of medical AI technology, alleviate the scarcity of medical resources and fulfil the medical and health demands of the public, it is crucial to improve the innovation behaviour of nurses through the use of medical XAI.

**Methods:** A cross-sectional survey was conducted involving 368 Chinese nurses working at tertiary and secondary hospitals in Anhui Province, Jiangsu Province, Zhejiang Province and Shanghai.

**Results:** Implementing medical XAI significantly enhanced the innovation behaviour of nurses. Anxiety and self-efficacy regarding AI mediated the connection between medical XAI and the innovation behaviour of nurses. Furthermore, the organizational ethical climate positively moderated the relationship between medical XAI and AI self-efficacy.

**Conclusion:** Medical XAI helps to enhance nurses' AI self-efficacy and reduce AI anxiety, thereby enhancing nurses' innovation behaviour. An organizational ethical climate enhances the positive relationship between medical XAI and AI self-efficacy.

**Implications for Nursing Management:** Organizations and technology developers must augment the study about XAI and the system design of human-centred AI technology. The organizations aim to enhance the education and training of nurses in AI, specifically focussing on boosting nurses' self-efficacy in utilizing AI technology. Moreover, they want to alleviate nurses' fear of new technological advancements. Hospital administrators and leaders develop strategies to address the ethical atmosphere inside their organization.

## 1. Introduction

Following numerous upgrades and reforms within China's medical sector, it is evident that the overall standard of healthcare has experienced notable enhancements [1, 2]. However, due to the imbalance in the supply and demand of nursing services, as well as the unreasonable allocation of medical resources, it becomes increasingly evident that the current traditional nursing service system could not meet the nursing needs in China [3–6]. The emergence and development of medical artificial intelligence (AI) has led to breakthroughs in exploring new models of medical service [7, 8]. In China, ophthalmology and dentistry are the two departments with a high prevalence of medical AI technology [9, 10]. For example, the first AI eye centre for cataracts has been established in Guangzhou, China [11]. AI is widely used in ophthalmology to assist in the diagnosis and therapeutic monitoring of ocular diseases such as diabetic retinopathy [12, 13], ocular tumours [14] and glaucoma [15]. AI is also widely used in dentistry to diagnose dental diseases such as caries and periapical inflammation [16, 17], formulate personalized treatment plans [18] and introduce dental implant robot [19], which improves the disadvantages of traditional oral diagnosis and treatment [20]. Nurses, as potential users of AI technology, are in a key position to shape and lead the development of modern AI in medical [21–23]. Nurses' innovation behaviour is beneficial to the popularization and application of medical AI technology, which could also drive the transformation and innovation of the nursing service in China [7, 8].

The concept of nurses' innovation behaviour pertains to the exploration and creation of novel technologies or methodologies to enhance health promotion, disease prevention and the quality of care [24, 25]. Labrague et al. [26] proposed the view that trainee nurses are willing to embrace technological advances and engage in innovation behaviour in nursing practice. To maximize the efficiency of medical AI in nursing, nurses contemplate methods to enhance or modify the implementation of medical AI in clinical and nursing practice [27, 28]. The barrier to nurses' involvement in AI research and collaborative design is a communication gap between nurses and technologists [29]. In addition, scholars have proposed that explainable artificial intelligence (XAI) should be conducted in light of the black-box nature to better understand the decision-making process of AI technology [30, 31]. The black-box property of AI technology refers to the fact that their decision-making processes and reasons are difficult to understand and explain [32]. Nurses must possess sufficient expertise in utilizing AI devices and effectively applying their knowledge in practice; however, an in-depth comprehension of the intricacies underlying the technology may not be a prerequisite [27, 33]. Thus, the explanation of AI should be modelled based on philosophy, psychology and the cognitive science of human interpretation [34] and cannot be explained solely through a technical perspective [35].

Arrieta et al. [36] proposed the concept of XAI from an audience perspective, which aims to make the decision-making and prediction processes within AI systems transparent and understandable. In the medical field, XAI has been defined as the extent to which nurses are able to understand the reasons for decisions made by AI systems [37, 38]. Furthermore, including medical personnel in developing and ultimately implementing healthcare AI technology can enhance their expertise rather than technology replacing them. This collaborative approach fosters a trustworthy relationship between medical professionals and technology, facilitating the advancement and widespread adoption of healthcare AI technology [27]. Researchers proposed to explore the XAI from the user's perspective in the technology development process to the application [30, 36, 39]. A limited number of studies exist investigating the potential impact of XAI on nurses' innovation behaviour [40].

According to the job demand–control model [41], medical AI technology changes nurses' work characteristics. It impacts their job requirements and control, affecting their psychological characteristics. Nurses with high self-efficacy have strong confidence and believe they can better cope with high job requirements, promoting innovation behaviour [8]. AI self-efficacy is defined as an individual's perception of their ability to use computers, rather than an individual's knowledge and understanding of computers [42, 43]. However, nurses with lower job control, to a large extent, have higher levels of stress and skill anxiety when their organizations implement new technology, refusing to use new technologies and avoid innovation behaviour. AI anxiety refers to the fear and anxiety that AI technology creates in individuals when it is beyond their control [7]. Social cognitive theory suggests that the environment influences individual cognition and behaviour [44]. The ethical climate of an organization is comprised of the collective knowledge and understanding of its members regarding what constitutes ethical conduct and how to resolve ethical dilemmas or problems [45], inevitably influencing the proactive behaviour and cognition of nurses [46–48].

This study explored the relationship between medical XAI and nurses' innovation behaviour, examining how AI self-efficacy and AI anxiety mediate this relationship. Additionally, the study investigated the moderating role of organizational ethical climate. By addressing these aspects, this research revealed how enhancing medical XAI can promote nurses' innovation behaviour, further advancing the integration of medical AI technology into nursing practice. It provided new insights for innovation in nursing management.

## 2. Research Hypothesis

### 2.1. XAI and Nurses' Innovation Behaviour

The present evaluation of medical XAI primarily evaluates the explainability of the model and application effect. The explainability takes accuracy, causality and stability as the measurement indicators, while the application takes the users' security, fairness and visualization as the evaluation indicators [49]. The essence of medical XAI is the interpretation of AI models developed from large amounts of intricate medical data [50] to make people understand how AI systems arrive at particular conclusions, recommendations and algorithms they rely on. Research has indicated that FATE (fairness, accountability, transparency and explainability) has a positive effect on the acceptance of algorithmic services, providing practical suggestions for the development of human-centred or responsible and trustworthy AI [51–53]. To measure XAI, the present study primarily adopts Shins' perspectives.

Nurses are likely to pay more attention to XAI, an important factor influencing the development and implementation of medical AI [28, 35, 54]. Social cognitive theory proposes that goals can elicit and direct motivational outcomes [55, 56]. Medical AI is highly explainable and can explain the foundation of its decisions, resulting in nurses' safety, responsibility and transparency, which improves nurses' trust in medical AI technology [30, 57]. With improving the cognition and trust in medical AI technology, nurses may be willing to adopt and utilize medical AI technology in order to better achieve their goals and, to some extent, inspire innovation behaviour [26]. As an applied discipline, nursing actively promotes pursuing and delivering innovative medical services and emphasizes innovation as an opportunity to generate progress [58]. A lack of explainability in medical AI or algorithms hinders nurses' understanding and acceptance, diminishing their trust in such systems [54, 59]. Nurses adopt traditional diagnosis and treatment modes, avoid taking risks and do not actively innovate and explore new medical service methods.

**Hypothesis 1 .**

Medical XAI positively affects nurses' innovation behaviour.

### 2.2. Mediating Role of AI Self-Efficacy Between Medical XAI and Nurses' Innovation Behaviour

AI self-efficacy refers to an individual's belief in using AI technology to achieve work goals [60]. A high sense of self-efficacy implies that individuals can perform various tasks and job requirements effectively. Individual confidence leads to better performance and effectiveness of the AI technology, motivating the user to approach job challenges with greater initiative. According to the job demand–control model, the introduction of medical AI technology increases the job demands on nurses, which represent stressors in the work environment such as time pressure and excessive workload [61, 62]. Job control refers to an individual's ability to control work and tasks [63]. Job demand–control theory puts forward the buffering hypothesis that improving the sense of job control can reduce the negative impact of high job demands [64]. When medical XAI improves, it helps nurses' control and use medical AI to mitigate the negative effects of high work demands. Therefore, when nurses' sense of control over medical AI is enhanced, it helps to improve nurses' sense of AI self-efficacy. A high level of medical XAI inspires nurses to believe that AI can assist in the making of accurate and valuable auxiliary decisions, helps them gain confidence in AI technology for medical AI diagnosis and treatment and strengthens their trust in AI, which in turn improves their comprehension of novel technologies and knowledge, their conviction in task completion and their self-assurance.

Moreover, nurses with high self-efficacy are confident in their ability to solve challenges and foster innovation using AI technology [7, 8]. They are open to experimenting with novel approaches and technologies to deliver superior medical services and treatment to patients. Furthermore, nurses with high self-efficacy are eager to engage in continuous learning and technological innovation and seek opportunities to solve clinical practical problems such as diagnosis, treatment and AI technology improvement [65]. Therefore, medical XAI helps to improve the self-efficacy of nurses and ultimately promote their innovation.

**Hypothesis 2 .**

AI self-efficacy plays a mediating role between medical XAI and nurses' innovation behaviour.

### 2.3. Mediating Role of AI Anxiety Between Medical XAI and Nurses' Innovation Behaviour

AI anxiety refers to an individual's irrational anxiety and emotional response, leading to behaviour such as avoidance of use and reflecting their fear and unease about AI beyond their control [66]. According to the addictive strain hypothesis proposed in the job demand–control model, employees working in high job demand, low job control environments have the most negative mental health and the highest stress levels [61, 67]. Although AI technology is rapidly being used in the medical field for its high accuracy, high efficiency and noncontact treatment, for nurses who lack knowledge and understanding of AI technology, the introduction of medical AI technology can lead to a rise in nurses' job demand and a decrease in job control. Nurses' job stress arises from the imbalance between job demand and job control [8]. Individuals in long-term high pressure will cause anxiety and other bad health state [68]. Research showed that more information and knowledge about AI technology can help users reduce their anxiety when facing new technologies [35, 69]. Certain concerns can be alleviated by engaging in transparent discussions about the ethics of AI in healthcare [70]. Medical XAI has the potential to aid nurses in understanding the underlying logic behind judgements made by AI algorithm models, partially reducing their anxiety associated with AI.

The limited comprehension of AI technology among nurses will impact their concern regarding their skill and expertise in medical AI [71], thus affecting their motivation to participate in innovation and their level of innovation ability. Furthermore, AI anxiety also includes concerns about technology reliability, privacy and security. A lack of motivation and enthusiasm for innovation behaviour, scepticism and resistance to technological change and a reluctance to attempt new AI technology solutions or alter established work processes may result from numerous technical anxieties among nurses [72]. If nurses can overcome AI anxiety and actively respond to new work requirements, they can adapt and accept AI technology and believe that these technologies can provide better medical care. The promotion and widespread adoption of technology among nurses is contingent upon user-friendliness [73]. These technologies can relieve nurses of tedious tasks, freeing them to devote more time and energy to core nursing work and tasks that promote nursing innovation [35].

**Hypothesis 3 .**

AI anxiety plays a mediating role between medical XAI and nurses' innovation behaviour

### 2.4. The Moderating Role of Organizational Ethical Climate

Organizational ethical climate pertains to the collective knowledge and understanding of its members regarding moral dilemmas and influences individuals' attitudes, beliefs and intentions towards moral issues. Furthermore, it profoundly impacts the ethical conduct and decision-making of the entire organization [74]. According to the social cognitive theory, individual activities are formed by interacting with three factors: individual cognitive characteristics, external environment and individual behaviour. Individuals will constantly regulate themselves by comparing their own behaviours with those advocated by the organizational ethical climate [55, 75]. The positive organizational ethical climate emphasizes the ability of nurses to provide a high level of medical care, as well as the ethical norms and professional principles that nurses should follow when making decisions [76–78]. An effective organizational ethical climate can give nurses access to resources and information by establishing medical XAI with principles of transparency, accountability, safety and fairness [52]. Adoption of medical AI helps nurses to provide a higher level of health care to their patients [79]. Therefore, in a positive organizational ethical climate, organizations prioritize the development of applications related to medical AI. Nurses are willing to continue to learn professional knowledge of AI technology to improve their awareness, application and innovation ability of medical AI [35, 80]. These nurses are optimistic about their ability to effectively implement medical AI technology to deliver successful diagnosis and treatment services. Consequently, a strong ethical climate within an organization enhances the positive correlation between medical XAI and nurses' AI self-efficacy.

**Hypothesis 4 .**

Organizational ethical climate has a moderating effect on medical XAI and AI self-efficacy.

**Figure 1:**
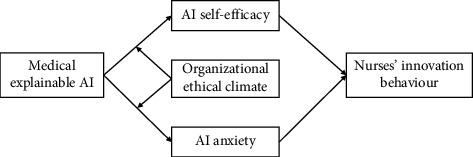
The hypothesized study model.

Conversely, medical personnel may develop confidence in and endorse the management and organization due to a robust ethical climate within the institution [39, 46]. Nurses find it more manageable to comprehend the decisions and outcomes of medical AI when they have access to detailed information and relevant explanations about medical AI [72]. Social cognitive theory emphasizes the individual's observation of the environment and imitation [44]. Nurses observe colleagues beforehand when they are sceptical about their ability to use medical AI. Observing the colleague successfully use a medical AI to perform a job task can reduce the observer's anxiety [81]. In an organizational ethical climate, information transfer and communication mechanisms ensure that nurses understand the explainability of healthcare AI systems and reduce their AI anxiety by eliminating the sense of uncertainty surrounding their decisions. The strong organizational ethical climate weakens the negative relationship between medical XAI and AI anxiety. Theoretical model of this study is presented in Figure 1.

**Hypothesis 5 .**

Organizational ethical climate has a moderating effect on medical XAI and AI anxiety.

## 3. Research Methods

### 3.1. Sample and Procedure

This study focused on two departments (ophthalmology and dentistry), the frontier fields of AI technology application in China's medical field. Due to the convenience and variety of image data acquisition in these departments, they were transformed into research departments, with nurses serving as subjects of the investigations. This study was conducted from May 2023 to July 2023, with nurses from private hospitals and the top three hospitals in Anhui Province, Jiangsu Province, Zhejiang Province and Shanghai. The requirements of the respondents in this study were junior professional titles, intermediate professional titles and licenced assistant physician titles. They are working on healthcare related to medical AI and have worked in the department for at least 6 months.

Common method bias (CMB) refers to the perceived covariation between predictor variables and calibration variables due to the same data sources or the same raters, the same measurement environment, the context of the research and the characteristics of the items [82]. To prevent the effects of CMB, this study conducted a two-stage investigation [82], with a 2-month interval between the two surveys. The first stage of the survey began in May 2023. The respondents who completed the survey left the last four digits of their mobile phone numbers as matching codes. The distribution of 550 questionnaires resulted in the retention of 523 valid questionnaires after excluding incomplete and invalid questionnaires. In July 2023, questionnaires were disseminated to respondents who had completed the initial phase of the investigation. After eliminating unqualified and incomplete responses, the two questionnaires were compared. The recovery rate for the 368 valid questionnaires that were gathered was 66.91%.

### 3.2. Measurements

The questionnaire used in this study consists of three parts. The first part explains that the questionnaire was used for academic research only and that the participants in the study participated voluntarily and anonymously, with an undertaking to keep the questionnaire data safe and to safeguard the privacy of the participants. The second part deals with the demographic characteristics of the participants (age, gender, education and clinical experience). The third section deals with the measurement of five variables, including medical XAI, AI self-efficacy, AI anxiety, organizational ethical climate and innovation behaviour. The variables were measured using reliable and mature scales developed. Likert's five-point scale was used, with one to five ranging from ‘*Strongly Disagree*' to ‘*Strongly Agree*'. For the initial scales that were in English, in order to minimize the impact of cultural and linguistic differences, a translation-back-translation method was used to convert the relevant scales into Chinese. In addition, the questionnaire items were appropriately adjusted according to the study context. Before formally distributing the questionnaires, three nurse leaders were asked to review the questionnaires to ensure that the internal logic of the questionnaires was smooth and the use of terminology was correct.

Medical XAI was adopted from the scale of explainable AI by Shin [51], including four items. The representative item was ‘I found medical AI are easily understandable'. The scale's reliability coefficient was 0.73.

AI self-efficacy was adopted from the scale of robot use self-efficacy in healthcare work by Turja et al. [83], including six items. The representative item was ‘I'm confident in my ability to learn how to use medical AI if they were to become part of my unit'. The scale's reliability coefficient was 0.84.

AI anxiety was measured from Huo et al. [7] four items. The representative item was ‘With the large scale of medical AI application, I am concerned that medical staff lose control in the process of medical services'. The scale's reliability coefficient was 0.75.

Innovation behaviour was measured from Bao et al. [24] 10 items. The representative items were ‘When working with medical AI, I will find the problems and be willing to solve them'. The reliability coefficient of the scale was 0.94.

Organizational ethical climate was measured from Vidaver-Cohen [84] five items. The representative item was ‘The hospital where I work will require medical personnel to adhere strictly to relevant ethical guidelines when introducing new technologies such as medical artificial intelligence'. In this study, the scale's reliability coefficient was 0.87.

### 3.3. Ethical Consideration

In the survey, an informed consent letter was sent to participants, explicitly stating that their involvement in the study was both voluntary and anonymous. After completing the survey, the collected data were securely stored to ensure the confidentiality of the participants. Our research complied with ethical norms and laws. The Science and Technology Research Ethics Committee of Anhui University of Science and Technology approved the protocol of this study (Ethical ID: LW-2023-001).

### 3.4. Data Analysis

The data analysis was conducted using Amos 26.0 and SPSS 26.0. First, this study used the Harman one-way test by SPSS 26.0 to prevent CMB may cause serious confusion in the research results and potentially misleading the conclusions [82]. All items of the variables were subjected to unrotated factor analysis. If the first principal component explained less than 40% of the variance was obtained, it means that there is no significant CMB.

Second, the reliability and validity tests are an important condition to ensure the accuracy of the results of data analysis. Before hypothesis testing, this study needs to evaluate the reliability and validity of variable items. The reliability denotes the degree of internal consistency and stability of the measurement instrument, which is used to examine the reliability of variable measurements. The validity refers to the degree to which a test indicator can accurately measure the variable to be measured, that is, the accuracy of measurement results. It reveals the relationship between variables and measurement items.

Among them, the reliability test is usually evaluated by Cronbach's *α* coefficient. When Cronbach's *α* coefficient is higher than 0.7, the model has high internal consistency [85, 86]. Confirmatory factor analysis (CFA) is a common method to test the discriminative validity. In addition, in order to compare actual data and model fit to meet validation statistical criteria, enhancing the explanatory power and reliability of the assessment model, CFA methods can also be tested [87]. Commonly used validity indicators include *χ*^2^/d*f* ≤ 3, RMSEA ≤ 0.08, CFI ≥ 0.90, TLI ≥ 0.90, NFI ≥ 0.90 and IFI ≥ 0.90 [88].

Further, Pearson was used to conduct a correlation analysis of the variables to test whether there is a multicollinearity problem between the variables in this study. When the correlation coefficient between variables exceeds 0.75, it is considered that the correlation between variables is high and there may be a multicollinearity problem [89, 90]. However, correlation analyses can only indicate whether there is a correlation between the variables and do not reveal the causal relationship between variables and the degree of its impact.

Finally, the hypotheses proposed, including main, mediating and moderating effects, are then tested when the reliability and validity of the measurement model meet the standard, and the data do not have serious CMB and multicollinearity problems.

## 4. Results

### 4.1. Demographic Characteristics

Table 1 presents the demographic characteristics of nurses, valid sample 368. Among them, the gender was female, accounting for 76.4%; most participants were less than 30 years old (82.6%), 71.7% had a bachelor's degree, 6.5% had a master's degree or above, 45.9% had less than 1 year of clinical experience, and 41.0% had 1–5 years of clinical experience.

### 4.2. CMB and CFA

Before commencing the investigation, this study implemented anonymity measures, including the hiding of variable names and the linguistic modification of questionnaire items, to minimize the influence of CMB. Nevertheless, it remained imperative to examine the potential bias. By employing Harman's single-factor test, we ascertained that the first common factor accounted for 37.39% of the variance in the total variables. This value is below the threshold of 40%, which signifies no obvious CMB in the research data.

In this study, CFA was used to examine the discriminant validity of the variables.  Table 2 shows that the five-factor model had the best fit (*χ*^2^/d*f* = 2.548, NFI = 0.902, IFI = 0.938, TLI = 0.923, CFI = 0.937, RMSEA = 0.065). All indexes were better than those of other models, indicating that the variables in this study had good discriminant validity.

### 4.3. Correlation Study

Table 3 shows the mean value, standard deviation and correlation among variables in this study. The data shown in Table 3 imply that medical XAI is positively correlated with AI self-efficacy (*r* = 0.430, *p* < 0.01), innovation behaviour (*r* = 0.338, *p* < 0.01) and negatively correlated with AI anxiety (*r* = −0.262, *p* < 0.01). AI self-efficacy was positively correlated with innovation behaviour (*r* = 0.409, *p* < 0.01), whereas AI anxiety was negatively correlated with innovation behaviour (*r* = −0.321, *p* < 0.01). These results provide a foundation for investigating the potential mediating effect of AI self-efficacy and AI anxiety on the relationship between medical XAI and nurses' innovation behaviour.

### 4.4. Hypothesis Test Results

**Figure 2:**
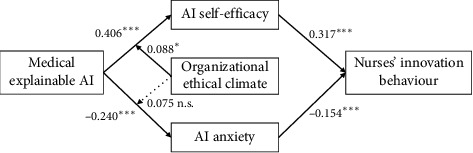
Structural equation model. *Note:*^∗^*p* < 0.05; ^∗∗∗^*p* < 0.001; n.s. means that the path is not significant.

Figure 2 presents the data after controlling for nurses' age, gender, education and clinical experience. Medical XAI positively impacts nurses' innovation behaviour (*β* = 0.319, *p* < 0.001), supporting Hypothesis 1. Furthermore, medical XAI had a positive impact on AI self-efficacy (*β* = 0.406, *p* < 0.001), and AI self-efficacy had a positive impact on nurses' innovation behaviour (*β* = 0.317, *p* < 0.001). Medical XAI had a negative effect on AI anxiety (*β* = −0.240, *p* < 0.001), and AI anxiety had a negative effect on nurses' innovation behaviour (*β* = −0.154, *p* < 0.001).

The bootstrap method was adopted to examine the mediating effect of AI self-efficacy and AI anxiety.  Table 4 summarizes the results. Mediator Path 1 is medical XAI ⟶ AI self-efficacy ⟶ innovation behaviour, which tested the mediating effect of AI self-efficacy on medical XAI and innovation behaviour. The indirect effect size was 0.093 with a 95% confidence interval [0.042, 0.146], excluding 0. The mediating effect of AI self-efficacy was significant, and Hypothesis 2 was verified. This result confirms that AI self-efficacy plays a significant mediating role in the relationship between medical XAI and innovation behaviour. This means that improving medical XAI can increase nurses' AI self-efficacy and thus promote innovation behaviour among nurses.

Similarly, Mediator Path 2 was medical XAI ⟶ AI anxiety ⟶ innovation behaviour, testing the mediating effect of AI anxiety between medical XAI and innovation behaviour. 95% confidence interval [0.003, 0.065], excluding 0, estimated the indirect effect size to be 0.031. The mediating effect of AI anxiety was found to be significant, confirming Hypothesis 3. This result confirms that AI anxiety plays a significant mediating role in the relationship between medical XAI and innovation behaviour. This means that it is possible to reduce nurses' AI anxiety and thus promote their innovation behaviour by improving medical XAI.

As shown in Figure 2, the interaction terms of medical XAI and organizational ethical climate had a significant positive effect on AI self-efficacy (*β* = 0.088, *p* < 0.05), assuming that Hypothesis 4 was verified. The interaction term of medical XAI and organizational ethical climate was not significantly related to AI anxiety (*β* = −0.075, n.s.), not supporting Hypothesis 5.

**Figure 3:**
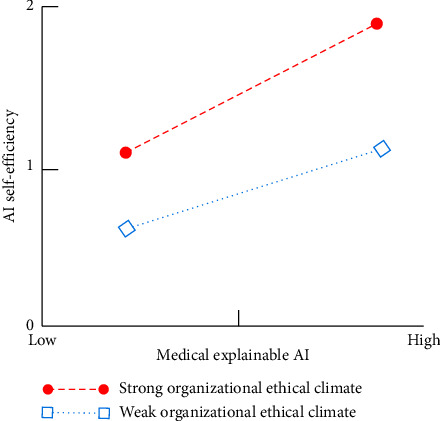
The moderating effect of organizational ethical climate on the relationship between medical XAI and AI self-efficacy.

In addition, based on the simple slope method recommended by Aiken and West [91], this study conducts the interaction effect diagram of the interaction of medical XAI with organizational ethical climate on AI self-efficacy.  Figure 3 shows that the positive effect of medical XAI on AI self-efficacy is higher when the organizational ethical climate is strong.

## 5. Discussion

Based on job demand–control theory and social cognitive theory, this study explores the path mechanisms of medical XAI influencing nurses' innovation behaviour and draws the following conclusions:

Medical XAI has a significant positive impact on nurses' innovation behaviour, supporting H1. The application of medical AI technology is mainly divided into auxiliary diagnosis and treatment technology and independent diagnosis and treatment technology [7]. The use of medical AI technology, such as image acquisition and surgical assistance, improves diagnostic efficiency and accuracy, reduces the work pressure of nurses and makes the assistant AI technology updated and popularized [15]. However, for some AI robots to independently complete online diagnosis and answer questions, the explainability of the technical algorithms cannot yet be accepted or recognized, and nursing the actual work needs matching is low. The effect of the application is not very good. The two medical AI technologies have different levels of explainability, with the more explainable medical AI technology being more acceptable and trusted by nurses, so there are differences in technology promotion and innovation development. The result of this study provides an answer to why there is a certain degree of divergence between the innovation and development of medical AI technology and why medical XAI promotes the innovation behaviour of nurses. That is, nursing managers should pay attention to the fact that medical XAI can influence nurses' innovation behaviour in nursing practice.

This study suggests that there are two different path mechanisms for the influence of medical XAI on nurse innovation behaviour. Specifically, the first pathway is that AI self-efficacy mediates the relationship between medical XAI and nurse innovation behaviour, supporting H2. Shin [73] proposed that medical XAI would enhance nurses' trust in AI technology and enhance nurses' confidence and intrinsic motivation to improve innovation. The core of AI self-efficacy is the belief that individuals can effectively use AI to accomplish their work [92]. Nurses can enhance the practicality of medical AI technology and improve technological innovation by offering developers feedback regarding its safety, accountability, transparency and explainability [36] and promote technological innovation. When medical XAI is at a high level, nurses perceive medical AI as simple and easy to understand, increasing nurses' confidence in utilizing medical AI and applying medical AI to solve problems in clinical practice. Because nurses believe they can overcome challenges and successfully apply these technologies. Nurses with high AI self-efficacy will hold a more open and inclusive attitude towards medical AI and are more willing to use the new technology to solve problems in clinical practice [43, 60, 92], thus promoting innovation behaviour.

The second pathway is that AI anxiety mediates the relationship between medical XAI and nurse innovation behaviour, supporting H3. According to the job demand–control model, the four combinations of job demand and job control affect employee creativity [93]. The combination of high job demand and low job control is more likely to lead to higher exhaustion and lower motivation [94]. It is challenging for nurses to identify these errors or biases due to the opacity of AI [95], which does not provide a foundation for overturning or correcting the decision. Low levels of medical XAI can become the job demand for nurses, especially when nurses are required to learn to utilize medical AI for more complex care tasks, putting nurses in a state of anxiety, which is consistent with a state of high job demand and low job control. Whereas anxiety as a poor mental leads to negative behaviour [96], it can reduce nurses' acceptance of medical AI as a new technology and they will be reluctant to engage in innovation behaviour related to medical AI. The results reveal that nurses can enhance their understanding of medical AI technology by learning relevant knowledge in medical XAI. This can increase self-efficacy in using medical AI technology and reduce AI anxiety, which help nurses make informed decisions in nursing practice. On the one hand, it promotes nurses' innovation behaviour, advancing the development and innovation of the nursing services industry. On the other hand, once nurses become proficient in medical AI technology, they can also assist developers in improving technology or algorithms, promoting the emergence of more advanced and user-friendly medical AI technology, thereby advancing nursing innovation and development. Additionally, the results also address the cognitive pathways explaining why medical AI technology has both positive and negative impacts [69, 97, 98].

Finally, the study introduced organizational ethical climate as a moderating variable. Organizational ethical climate positively moderated the relationship between medical XAI and AI self-efficacy, supporting H4. However, the moderating effect was not significant in the relationship between medical XAI and AI anxiety, not supporting H5. This may be because the application and promotion of medical AI technology are still developing, and nurses are not too worried about the technology. Social cognitive theory emphasizes the individual's observation of the environment and imitation [44]. Fairness, accountability and transparency will be emphasized in the positive ethical climate of the organization [99, 100]. This positive environment enhances the importance of medical AI in organizations and makes organizations inclined to introduce medical AI devices or technologies with high explainability. An organizational ethical climate can help nurses develop a correct understanding of medical AI technology, enabling them to rigorously monitor AI in nursing practice. Such behaviour aids in preventing individuals from evading medical responsibilities, enhancing trust in AI and reducing the negative impacts of new technologies. This study indicates that nursing management personnel can introduce the implementation of ethics education and training systems related to AI technology within organizations, leading to a significant improvement in nurses' AI self-efficacy. The organizational ethical climate as a moderating variable can resolve doubts for organizations about how and what to intervene. Establishing a positive organizational ethical climate can not only facilitate the integration of medical AI technology into nursing practice but also promote innovation in the nursing services and improve the patient care experience.

## 6. Conclusions

This study provides new insights into the factors that promote nurses' innovation behaviour. Overall, this study explored the dual-path model of the impact of medical XAI on nurses' innovation behaviour based on the job demand–control model and social cognitive theory, and this study examined how medical XAI affects nurses' innovation behaviour. It can be concluded that AI self-efficacy and AI anxiety mediated the relationship between medical XAI and nurses' innovation behaviour separately. As a moderating variable, organizational ethical climate moderates the effect of medical XAI on nurses' AI self-efficacy. The findings suggest that the explainability of medical AI in terms of fairness, accountability and transparency can be enhanced from the cross-disciplinary perspective of humanities and social sciences, which can provide new ideas for the development of medical AI technology and system design, and provide theoretical support for promoting and training nurses' innovation behaviour. In addition, strengthening the promotion and application of medical AI technology and enriching new nursing service models can provide new ideas for solving the problems of shortage of medical personnel and unequal distribution of medical resources.

### 6.1. Implications for Nursing Management

This study explores the relationship between medical XAI and the innovation behaviour of Chinese nurses, with practical implications for the nurse community, organizational managers, student nurse education schools and the field of medical AI. Details are shown as follows:

1. Cross-disciplinary collaboration is carried out to encourage nurses' participation in the medical AI design. There is a need to empower nurses to collaborate with technologists, doctors and other healthcare professionals to jointly develop and evaluate AI solutions. This interdisciplinary collaboration helps ensure the applicability of AI technology devices. For example, it is suggested that organizations and technology developers need to enhance the understanding and research of medical XAI from the perspective of humanities and social sciences for system design of human-centred medical AI technology. In healthcare, medical XAI is critical for nurses, patients and other stakeholders, who must understand how AI technology makes decisions and what data are used to make predictions or diagnoses [39, 73]. Organizations and technology developers need to prioritize the design of user-friendly interfaces that facilitate nurses' understanding of medical AI outcomes and decisions. We ensure that the decision-making process of the AI system is transparent, and establish feedback mechanisms so that nurses can report and discuss issues and challenges in practical use. Furthermore, it is crucial to establish traceability and reviewability mechanisms within the decision-making process of medical AI. This will enable healthcare workers to verify the reliability of the system's decisions and hold it accountable for the results that it produces. Enhancing the legitimacy and acceptability of medical AI technology is crucial for fostering its integration into clinical practice.
2. Enhance nurse education and training. Organizations or societies should provide ongoing education on medical AI technology to enable nurses to understand and utilize these tools, enhance nurses' self-efficacy and reduce nurses' anxiety and concerns about new technology. Training should include how to explain the AI decision-making process and effectively use these technologies in daily nursing practice such as encompassing fundamental information, practical applications, technical principles and operational procedures about medical AI. Training programs effectively enhance nurses' comprehension of the potential and prospects of medical AI [29, 101]. These programs also equip nurses with the necessary skills and knowledge to effectively utilize AI technology [26]. Organizations can utilize practical scenarios to facilitate nurses' understanding and recognition of the practical implications of AI technology and bolster their confidence in and self-assurance regarding using such technology. Nurses can better adapt and utilize medical AI technology and reduce anxiety with continuous support and guidance from relevant medical organizations.
3. The ethical issues of medical AI should be paid attention to, and a positive organizational ethical climate should be established. The organizations strengthen research on medical AI ethics and set up a special research institute or ethics committee responsible for researching and regulating issues related to medical AI ethics. These institutions may comprise professionals and academics with diverse areas of expertise, including but not limited to medicine, ethics, law and computer science. They engage in interdisciplinary collaboration to incorporate various perspectives and interests, aiming to offer more efficient resolutions for ethical dilemmas in research and response [34, 102]. It is essential to develop a framework and guiding principles for ethical issues in medical AI through research and discussion, which can cover data use, algorithmic fairness, transparency, privacy protection, accountability and other aspects of medical AI technology to guide the development, application and regulation of medical AI technology. The organizations establish contact with international partners to share experiences and research results. Countries and regions may have different concerns and research on the ethical issues of medical AI [98]. By fostering international collaboration, we can advance global cooperation in investigating and resolving ethical concerns associated with medical AI and gain knowledge from one another [103, 104].

### 6.2. Limitations

The present study has some limitations. First, the study focused on dental and ophthalmology nurses as the research participants and did not specifically address the impact of medical AI implementation and promotion across various departments. To address this limitation, future research could investigate the different departments of nurses using healthcare AI as a way to improve the representativeness of the data and the generalizability of the findings. Second, Huo et al. [105] have categorized medical AI into auxiliary and autonomous technologies based on the classification of its applications. Subsequent research endeavours may explore the varying effects of different types of medical AI on nurses' innovation behaviour. Third, the research employed solely a questionnaire survey as the data collection method, which poses limitations in obtaining comprehensive feedback from nurses regarding the actual implementation of medical AI. To address this limitation, future investigations may explore using case studies and experimental methods in the later stages of the study.

## Figures and Tables

**Table 1:** Nurse's characteristics (*N* = 368).

**Variables**	**Frequency (f)**	**Percentage (%)**
Gender		
Male	87	23.6
Female	281	76.4
Age		
20 or less	85	23.1
21–30	219	59.5
31 to 40	39	10.6
41 to 50	23	6.3
50 or higher	2	0.5
Education level		
High school	54	14.7
Junior college	26	7.1
University	264	71.7
Master and above	24	6.5
Clinical experience		
1 or less	169	45.9
1–5	151	41.0
6–10	39	10.6
11–20	6	1.6
21 or higher	3	0.8

**Table 2:** Results of confirmatory factor analysis (CFA).

**Model**	**Variables**	**χ** ^2^ **/df**	**NFI**	**IFI**	**TLI**	**CFI**	**RMSEA**
Five-model	XAI, AISE, AIA, IB, OEC	2.548	0.902	0.938	0.923	0.937	0.065
Four-model	XAI, AISE + AIA, IB, OEC	3.547	0.859	0.894	0.874	0.893	0.083
Three-model	XAI + OEC, AISE + AIA, IB	5.144	0.79	0.824	0.794	0.823	0.106
Two-model	XAI + OEC + AISE + AIA, IB	6.304	0.739	0.771	0.737	0.769	0.120
One-model	XAI + OEC + AISE + AIA + IB	9.468	0.605	0.631	0.580	0.629	0.152

Abbreviations: *χ*^2^, chi-square fit statistics; AIA, AI anxiety; AISE, AI self-efficacy; CFI, comparative fit index; d*f*, degrees of freedom; IB, innovation behaviour; IFI, incremental fit indices; NFI, normed fit index; OEC, organizational ethical climate; RMSEA, root-mean-square error of approximation; TLI, Tucker–Lewis index; XAI, medical explainable artificial intelligence.

**Table 3:** Means, standard deviations and correlations among variables (*n* = 368).

	**M**	**SD**	**1**	**2**	**3**	**4**	**5**
1 XAI	3.627	0.737	**0.722**				
2 AISE	3.583	0.676	0.430^∗∗^	**0.756**			
3 AIA	3.566	0.714	0.262^∗∗^	0.471^∗∗^	**0.708**		
4 IB	3.543	0.683	0.338^∗∗^	0.409^∗∗^	0.321^∗∗^	**0.781**	
5 OEC	3.766	0.698	0.350^∗∗^	0.582^∗∗^	0.451^∗∗^	0.401^∗∗^	**0.789**

*Note*: The bold values indicate the average variance extracted (AVE) values.

Abbreviations: AIA, AI anxiety; AISE, AI self-efficacy; IB, innovation behaviour; M, mean; OEC, organizational ethical climate; SD, standard deviation; XAI, medical explainable artificial intelligence.

^∗∗^ *p* < 0.01.

**Table 4:** Direct and indirect effects.

**Dependent variables**	**Paths**	**Effect size**	**Standard error**	**95% confidence interval**	
				**Upper limit**	**Lower limit**
Innovation behaviour	Total effect	0.296	0.046	0.205	0.386
	Direct effects	0.172	0.048	0.077	0.266
	Indirect effects	0.124	0.028	0.071	0.180
	Mediator Path 1: XAI ⟶ AI self-efficacy ⟶ innovation behaviour	0.093	0.027	0.042	0.146
	Mediator Path 2: XAI ⟶ AI anxiety ⟶ innovation behaviour	0.031	0.016	0.003	0.065

Abbreviation: XAI, medical explainable artificial intelligence.

## Data Availability

The data that support the findings of this study are available from the corresponding author upon reasonable request.

## References

[1] Sun W., Zhu H., Zhang L. (2023). Do Medical Alliances Truly Work? Perspectives on Health Service Utilisation Among Outpatients With Chronic Diseases in Shanghai, China. *Australian Journal of Primary Health*.

[2] Lin Y., Li L., Liu B. (2024). Assessing the Price Levels of Medical Service and Influential Factors: Evidence From China. *BMC Public Health*.

[3] Lin J., Zhou J., Wang L. (2020). Health Care Reform in China From the Perspective of Physicians. *BioScience Trends*.

[4] Wang S., Tao Q. (2021). Analysis of the Efficiency of China’s Medical Service Resources Under the Background of Hierarchical Medical Policy. *Iranian Journal of Public Health*.

[5] Fan V., Guo M., Hou J., Talagi D., Ke Y., Wang W. (2022). Factors Associated With Selection of Practice in Primary Care and Rural Health Among Medical and Nursing Students in China. *Australian Journal of Primary Health*.

[6] Wu W., Long S., Cerda A. A., Garcia L. Y., Jakovljevic M. (2023). Population Ageing and Sustainability of Healthcare Financing in China. *Cost Effectiveness and Resource Allocation*.

[7] Huo W., Yuan X., Li X., Luo W., Xie J., Shi B. (2023). Increasing Acceptance of Medical AI: The Role of Medical Staff Participation in AI Development. *International Journal of Medical Informatics*.

[8] Li N., Zhang L., Li X., Lu Q. (2022). Moderated Role of Social Support in the Relationship Between Job Strain, Burnout, and Organizational Commitment Among Operating Room Nurses: A Cross-Sectional Study. *International Journal of Environmental Research and Public Health*.

[9] Zheng B., Wu M. N., Zhu S. J. (2021). Attitudes of Medical Workers in China Toward Artificial Intelligence in Ophthalmology: A Comparative Survey. *BMC Health Services Research*.

[10] Shan T., Tay F. R., Gu L. (2021). Application of Artificial Intelligence in Dentistry. *Journal of Dental Research*.

[11] Ruamviboonsuk P., Cheung C. Y., Zhang X., Raman R., Park S. J., Ting D. S. W. (2020). Artificial Intelligence in Ophthalmology: Evolutions in Asia. *Asia-Pacific Journal of Ophthalmology*.

[12] Dai L., Wu L., Li H. (2021). A Deep Learning System for Detecting Diabetic Retinopathy Across the Disease Spectrum. *Nature Communications*.

[13] Hao S., Liu C., Li N. (2022). Clinical Evaluation of AI-Assisted Screening for Diabetic Retinopathy in Rural Areas of Midwest China. *PLoS One*.

[14] Bi S., Chen R., Zhang K. (2020). Differentiate Cavernous Hemangioma From Schwannoma With Artificial Intelligence (AI). *Annals of Translational Medicine*.

[15] Chen A., Yang T., Ma J., Lu Y. (2023). Employees’ Learning Behavior in the Context of AI Collaboration: A Perspective on the Job Demand-Control Model. *Industrial Management & Data Systems*.

[16] Zhu Y., Xu T., Peng L. (2022). Faster-RCNN Based Intelligent Detection and Localization of Dental Caries. *Displays*.

[17] Li S., Liu J., Zhou Z. (2022). Artificial Intelligence for Caries and Periapical Periodontitis Detection. *Journal of Dentistry*.

[18] Xie B., Xu D., Zou X. Q., Lu M. J., Peng X. L., Wen X. J. (2024). Artificial Intelligence in Dentistry: A Bibliometric Analysis From 2000 to 2023. *Journal of Dental Science*.

[19] Yan B., Zhang W., Cai L. (2022). Optics-Guided Robotic System for Dental Implant Surgery. *Chinese Journal of Mechanical Engineering*.

[20] Grischke J., Johannsmeier L., Eich L., Griga L., Haddadin S. (2020). Dentronics: Towards Robotics and Artificial Intelligence in Dentistry. *Dental Materials*.

[21] McGrow K. (2019). Artificial Intelligence: Essentials for Nursing. *Nursing*.

[22] Huang C., Wang J., Wang S., Zhang Y. (2023). A Review of Deep Learning in Dentistry. *Neurocomputing*.

[23] Huang X. M., Yang B. F., Zheng W. L. (2022). Cost-Effectiveness of Artificial Intelligence Screening for Diabetic Retinopathy in Rural China. *BMC Health Services Research*.

[24] Bao L., Wang L., Zhang Y. Q. (2012). Development and Analysis of Reliability and Validity of Nurse Innovative Behavior Scale. *Journal of Shanghai Jiaotong University*.

[25] Li X., Cheng M., Xu J. (2022). Leaders’ Innovation Expectation and Nurses’ Innovation Behaviour in Conjunction With Artificial Intelligence: The Chain Mediation of Job Control and Creative Self‐efficacy. *Journal of Nursing Management*.

[26] Labrague L. J., Aguilar-Rosales R., Yboa B. C., Sabio J. B., de Los Santos J. A. (2023). Student Nurses’ Attitudes, Perceived Utilization, and Intention to Adopt Artificial Intelligence (AI) Technology in Nursing Practice: A Cross-Sectional Study. *Nurse Education in Practice*.

[27] Matulionyte R., Nolan P., Magrabi F., Beheshti A. (2022). Should AI-Enabled Medical Devices be Explainable?. *International Journal of Law and Info Technology*.

[28] Vilone G., Longo L. (2021). Notions of Explainability and Evaluation Approaches for Explainable Artificial Intelligence. *Information Fusion*.

[29] Buchanan C., Howitt M. L., Wilson R., Booth R. G., Risling T., Bamford M. (2020). Predicted Influences of Artificial Intelligence on the Domains of Nursing: Scoping Review. *JMIR Nursing*.

[30] Ali S., Abuhmed T., El-Sappagh S. (2023). Explainable Artificial Intelligence (XAI): What we Know and What is Left to Attain Trustworthy Artificial Intelligence. *Information Fusion*.

[31] Kaur D., Uslu S., Rittichier K. J., Durresi A. (2022). Trustworthy Artificial Intelligence: A Review. *ACM Computing Surveys*.

[32] Kök I., Okay F. Y., Muyanli Ö., Özdemir S. (2023). Explainable Artificial Intelligence (Xai) for Internet of Things: A Survey. *IEEE Internet of Things Journal*.

[33] Kerasidou C. X., Kerasidou A., Buscher M., Wilkinson S. (2022). Before and Beyond Trust: Reliance in Medical AI. *Journal of Medical Ethics*.

[34] Miller T. (2018). Explanation in Artificial Intelligence: Insights From the Social Sciences. *Artificial Intelligence*.

[35] Liu C. F., Chen Z. C., Kuo S. C., Lin T. C. (2022). Does AI Explainability Affect Physicians’ Intention to Use AI?. *International Journal of Medical Informatics*.

[36] Arrieta A. B., Díaz-Rodríguez N., Del Ser J. (2020). Explainable Artificial Intelligence (XAI): Concepts, Taxonomies, Opportunities and Challenges Toward Responsible AI. *Information Fusion*.

[37] Borys K., Schmitt Y. A., Nauta M. (2023). Explainable AI in Medical Imaging: An Overview for Clinical Practitioners-Saliency-Based XAI Approaches. *European Journal of Radiology*.

[38] Martikainen S., Kaipio J., Lääveri T. (2020). End-User Participation in Health Information Systems (HIS) Development: Physicians’ and Nurses’ Experiences. *International Journal of Medical Informatics*.

[39] Meske C., Bunde E., Schneider J., Gersch M. (2022). Explainable Artificial Intelligence: Objectives, Stakeholders, and Future Research Opportunities. *Information Systems Management*.

[40] Yildirim N., Zlotnikov S., Sayar D. (2024). Sketching AI Concepts With Capabilities and Examples: AI Innovation in the Intensive Care Unit. https://arxiv.org/abs/2402.13437.

[41] Karasek R. (1989). The Political Implications of Psychosocial Work Redesign: A Model of the Psychosocial Class Structure. *International Journal of Health Services*.

[42] White G. L., Shah J. R., Cook J. R., Mendez F. (2008). Relationship Between Information Privacy Concerns and Computer Self-Efficacy. *International Journal of Technology and Human Interaction*.

[43] Rahman M. S., Ko M., Warren J., Carpenter D. (2016). Healthcare Technology Self-Efficacy (HTSE) and Its Influence on Individual Attitude: An Empirical Study. *Computers in Human Behavior*.

[44] Bandura A. (1999). Social Cognitive Theory: An Agentic Perspective. *Asian Journal of Social Psychology*.

[45] Victor G., Bélisle-Pipon J. C., Ravitsky V. (2023). Generative AI, Specific Moral Values: A Closer Look at ChatGPT’s New Ethical Implications for Medical AI. *The American Journal of Bioethics*.

[46] Sand M., Durán J. M., Jongsma K. R. (2022). Responsibility Beyond Design: Physicians’ Requirements for Ethical Medical AI. *Bioethics*.

[47] Wang Y. Y., Wang Y. S. (2022). Development and Validation of an Artificial Intelligence Anxiety Scale: An Initial Application in Predicting Motivated Learning Behavior. *Interactive Learning Environments*.

[48] Siau K., Wang W. (2020). Artificial Intelligence (AI) Ethics: Ethics of AI and Ethical AI. *Journal of Database Management*.

[49] Holzinger A., Dehmer M., Emmert-Streib F. (2022). Information Fusion as an Integrative Cross-Cutting Enabler to Achieve Robust, Explainable, and Trustworthy Medical Artificial Intelligence. *Information Fusion*.

[50] Johnson K. W., Torres Soto J., Glicksberg B. S. (2018). Artificial Intelligence in Cardiology. *Journal of the American College of Cardiology*.

[51] Shin D. (2021). The Effects of Explainability and Causability on Perception, Trust, and Acceptance: Implications for Explainable AI. *International Journal of Human-Computer Studies*.

[52] Schoonderwoerd T. A. J., Jorritsma W., Neerincx M. A., van den Bosch K. (2022). Human-Centered XAI: Developing Design Patterns for Explanations of Clinical Decision Support Systems. *International Journal of Human-Computer Studies*.

[53] Ding W., Abdel-Basset M., Hawash H., Ali A. M. (2022). Explainability of Artificial Intelligence Methods, Applications and Challenges: A Comprehensive Survey. *Information Sciences*.

[54] Hatherley J. J. (2020). Limits of Trust in Medical AI. *Journal of Medical Ethics*.

[55] Bandura A. (1986). *Social Foundations of Thought and Action*.

[56] Zimmerman B. J., Schunk D. H., DiBenedetto M. K. (2015). A Personal Agency View of Self-Regulated Learning. *Self-Concept, Motivation and Identity: Underpinning Success With Research and Practice*.

[57] Albahri A. S., Duhaim A. M., Fadhel M. A., Alnoor A., Baqer N. S. (2023). A Systematic Review of Trustworthy and Explainable Artificial Intelligence in Healthcare: Assessment of Quality, Bias Risk, and Data Fusion. *Information Fusion*.

[58] Felzmann H., Villaronga F. E., Lutz C. ., amò-Larrieux A. (2019). Transparency You Can Trust: Transparency Requirements for Artificial Intelligence Between Legalnorms and Contextual Concerns. *Big Data & Society*.

[59] Durán J. M., Jongsma K. R. (2021). Who is Afraid of Black Box Algorithms? On the Epistemological and Ethical Basis of Trust in Medical AI. *Journal of Medical Ethics*.

[60] Latikka R., Turja T., Oksanen A. (2019). Self-Efficacy and Acceptance of Robots. *Computers in Human Behavior*.

[61] Karasek R. A. (1979). Job Demands, Job Decision Latitude, and Mental Strain: Implications for Job Redesign. *Administrative Science Quarterly*.

[62] Ruokangas S. M., Weiste E., Ervasti J., Oksanen T., Nieminen P. (2022). Job Demands and Job Control Among Occupational Therapists in Public Sector in Finland. *Scandinavian Journal of Occupational Therapy*.

[63] Chen D., Ran E. A., Tan T. F. (2023). Applications of Artificial Intelligence and Deep Learning in Glaucoma. *Asia-Pacific Journal of Ophthalmology*.

[64] Dutheil F., Pereira B., Bouillon-Minois J. B. (2022). Validation of Visual Analogue Scales of Job Demand and Job Control at the Workplace: A Cross-Sectional Study. *BMJ Open*.

[65] Quinn T. P., Senadeera M., Jacobs S., Coghlan S., Le V. (2021). Trust and Medical AI: The Challenges We Face and the Expertise Needed to Overcome Them. *Journal of the American Medical Informatics Association*.

[66] Johnson D. G., Verdicchio M. (2017). AI Anxiety. *Journal of the Association for Information Science and Technology*.

[67] Gameiro M., Chambel M. J., Carvalho V. S. (2020). A Person-Centered Approach to the Job Demands-Control Model: A Multifunctioning Test of Addictive and Buffer Hypotheses to Explain Burnout. *International Journal of Environmental Research and Public Health*.

[68] Baron R. A., Franklin R. J., Hmieleski K. M. (2016). Why Entrepreneurs Often Experience Low, Not High, Levels of Stress: The Joint Effects of Selection and Psychological Capital. *Journal of Management*.

[69] Mirbabaie M., Brünker F., Möllmann N. R., Stieglitz S. (2021). The Rise of Artificial Intelligence-Understanding the AI Identity Threat at the Workplace. *Electronic Markets*.

[70] Stokes F., Palmer A. (2020). Artificial Intelligence and Robotics in Nursing: Ethics of Caring as a Guide to Dividing Tasks Between AI and Humans. *Nursing Philosophy*.

[71] Tjoa E., Guan C. (2020). A Survey on Explainable Artificial Intelligence (Xai): Toward Medical Xai. *IEEE Transactions on Neural Networks and Learning Systems*.

[72] Lötsch J., Kringel D., Ultsch A. (2021). Explainable Artificial Intelligence (XAI) in Biomedicine: Making AI Decisions Trustworthy for Physicians and Patients. *BioMedInformatics*.

[73] Shin D. (2020). User Perceptions of Algorithmic Decisions in the Personalized AI System: Perceptual Evaluation of Fairness, Accountability, Transparency, and Explainability. *Journal of Broadcasting & Electronic Media*.

[74] Victor B., Cullen J. B. (1988). The Organizational Bases of Ethical Work Climates. *Administrative Science Quarterly*.

[75] Domino M. A., Wingreen S. C., Blanton J. E. (2015). Social Cognitive Theory: The Antecedents and Effects of Ethical Climate Fit on Organizational Attitudes of Corporate Accounting Professionals—A Reflection of Client Narcissism and Fraud Attitude Risk. *Journal of Business Ethics*.

[76] Dziurka M., Ozdoba P., Olson L. (2022). Hospital Ethical Climate Survey-Selected Psychometric Properties of the Scale and Results Among Polish Nurses and Midwives. *BMC Nursing*.

[77] Teymoori E., Rahmani V., Fereidouni A., Khachian A., Hannani S. (2022). Ethical Climate of the Operating Room From the Perspective of the Surgical Team and Its Relationship With Organizational Culture and Organizational Commitment. *Perioperative Care and Operating Room Management*.

[78] Essex R., Thompson T., Evans T. R. (2023). Ethical Climate in Healthcare: A Systematic Review and Meta-Analysis. *Nursing Ethics*.

[79] Raymond L., Castonguay A., Doyon O., Paré G. (2022). Nurse Practitioners’ Involvement and Experience With AI-Based Health Technologies: A Systematic Review. *Applied Nursing Research*.

[80] Kempt H., Heilinger J. C., Nagel S. K. (2022). Relative Explainability and Double Standards in Medical Decision-Making: Should Medical AI be Subjected to Higher Standards in Medical Decision-Making Than Doctors?. *Ethics and Information Technology*.

[81] Schunk D. H., DiBenedetto M. K. (2020). Motivation and Social Cognitive Theory. *Contemporary Educational Psychology*.

[82] Podsakoff P. M., MacKenzie S. B., Lee J. Y., Podsakoff N. P. (2003). Common Method Biases in Behavioral Research: A Critical Review of the Literature and Recommended Remedies. *Journal of Applied Psychology*.

[83] Turja T., Rantanen T., Oksanen A. (2019). Robot Use Self-Efficacy in Healthcare Work (RUSH): Development and Validation of a New Measure. *AI & Society*.

[84] Vidaver-Cohen D. (1998). Moral Climate in Business Firms: A Conceptual Framework for Analysis and Change. *Journal of Business Ethics*.

[85] Chin W. W. (1998). The Partial Least Squares Approach to Structural Equation Modeling. *Modern methods for business research*.

[86] Charter R. A. (2001). Testing the Equality of Two or More Split-Half Reliability Coefficients. *Psychological Reports*.

[87] Cheung G. W., Rensvold R. B. (2002). Evaluating Goodness-of-Fit Indexes for Testing Measurement Invariance. *Structural Equation Modeling*.

[88] Hu L. T., Bentler P. M. (1999). Cutoff Criteria for Fit Indexes in Covariance Structure Analysis: Conventional Criteria Versus New Alternatives. *Structural Equation Modeling: A Multidisciplinary Journal*.

[89] Cote J. A., Buckley M. R. (1987). Estimating Trait, Method, and Error Variance: Generalizing across 70 Construct Validation Studies. *Journal of Marketing Research*.

[90] Grewal R., Cote J. A., Baumgartner H. (2004). Multicollinearity and Measurement Error in Structural Equation Models: Implications for Theory Testing. *Marketing Science*.

[91] Aiken L. S., West S. G. (1991). *Multiple Regression: Testing and Interpreting Interactions*.

[92] Latikka R., Savela N., Koivula A., Oksanen A. (2021). Attitudes Toward Robots as Equipment and Coworkers and the Impact of Robot Autonomy Level. *International Journal of Social Robotics*.

[93] Du Y., Xiao M., Li Z., Shen Y. (2023). Effect of Job Demands-Control Match on Employee Creativity: Perspective of Match in the Context of Job Design. *Current Psychology*.

[94] Mauno S., Mäkikangas A., Kinnunen U. (2016). A Longitudinal Person-Centred Approach to the Job Demands-Control Model. *European Journal of Work & Organizational Psychology*.

[95] Vo V., Chen G., Aquino Y. S. J., Carter S., Do Q., Woode M. E. (2023). Multi-stakeholder preferences for the use of artificial intelligence in healthcare: A systematic review and thematic analysis. *Social Science & Medicine*.

[96] Wemken G., Janurek J., Junker N. M., Häusser J. A. (2021). The Impact of Social Comparisons of Job Demands and Job Control on Well‐being. *Applied Psychology: Health and Well‐Being*.

[97] Grundner L., Neuhofer B. (2021). The Bright and Dark Sides of Artificial Intelligence: A Futures Perspective on Tourist Destination Experiences. *Journal of Destination Marketing & Management*.

[98] Wirtz B. W., Weyerer J. C., Sturm B. J. (2020). The Dark Sides of Artificial intelligence: An Integrated AI Governance Framework for Public Administration. *International Journal of Public Administration*.

[99] Borhani F., Jalali T., Abbaszadeh A., Haghdoost A. (2014). Nurses’ Perception of Ethical Climate and Organizational Commitment. *Nursing Ethics*.

[100] Yazdanmehr A., Jawad M., Benbunan-Fich R., Wang J. (2024). The Role of Ethical Climates in Employee Information Security Policy Violations. *Decision Support Systems*.

[101] Hatherley J., Sparrow R., Howard M. (2022). The Virtues of Interpretable Medical Artificial Intelligence. *Cambridge Quarterly of Healthcare Ethics*.

[102] Amann J., Blasimme A., Vayena E., Frey D., Madai V. I. (2020). Explainability for Artificial Intelligence in Healthcare: A Multidisciplinary Perspective. *BMC Medical Informatics and Decision Making*.

[103] Robinson S. C. (2020). Trust, Transparency, and Openness: How Inclusion of Cultural Valusshapes Nordic National Public Policy Strategies for Artificial Intelligence (AI). *Technology in Society*.

[104] Zhang J., Zhang Z. M. (2023). Ethics and Governance of Trustworthy Medical Artificial Intelligence. *BMC Medical Informatics and Decision Making*.

[105] Huo W., Zheng G., Yan J., Sun L., Han L. (2022). Interacting With Medical Artificial Intelligence: Integrating Self-Responsibility Attribution, Human-Computer Trust, and Personality. *Computers in Human Behavior*.

